# Sewing Machines and Health

**Published:** 1871-02

**Authors:** 


SEWING MACHINES AND HEALTH.—The ordinary sewing machine is often productive of ill health, from the cramped and unequal action it gives to the muscles of the body. Persons who use them should take care to restore the balance to the circulation before it becomes fixed and chronic, otherwise much harm will result. We hope that some bright inventor will try to invent a cheap electrical engine to run sewing machines. They would be a great blessing to women. It is said that a treadle has recently been invented that does not hurt the operator, but we have not yet had the pleasure of seeing it.—*Herald of Health.*

